# Supplementary material for: Severe leptospirosis in tropical Australia: Optimising intensive care unit management to reduce mortality
Source: PLoS Negl Trop Dis. 2019 Dec 2;13(12):e0007929. doi: 10.1371/journal.pntd.0007929 (PMC6907868; doi:10.1371/journal.pntd.0007929)
Supplement: Attachment — Submitted filename: 071119LeptoCL.pdf [file pntd.0007929.s002.pdf]

Queensland Government

Department of Medicine

Cairns & Hinterland Hospital & Health Service
Cairns Hospital, 1st Floor Block B
PO Box 902 CAIRNS QLD 4870
Ph: (07) 4226 8084 FAX: (07) 4226 8985

Dr Simon Smith
Infectious Diseases Physician
11/11/2019

Dear Prof. Joseph M. Vinetz & A/Prof. Melissa J. Caimano,

Re: PNTD-D-19-01625

Thank you for fully evaluating our manuscript "Severe leptospirosis in tropical Australia: optimising intensive care unit management to reduce mortality" for publication in PLOS Neglected Tropical Diseases. Please find below a detailed response to the reviewer’s comments.

Reviewer #1: The authors comment that corticosteroids therapy in these patients is indicated to toxin-mediated vasculitis or exaggerated host immune response (page 17, line 354). Experimental study demonstrated that in leptospirosis a non cardiogenic pulmonary edema may occur consequent to a reduction in expression of ENaC transporter in luminal membrane of the alveolar epithelial cells, since corticosteroids increases the expression of this transporter, it may be another mechanism that contributes to the treatment of ARDS in leptospirosis.

The authors would like to thank Reviewer 1 for their feedback and for highlighting this important point. This has been added to the manuscript after page 17, line 354 and a reference provided.

How many patients presented AKI with moderated to severe ARDS?

A total of 35 patients presented with both acute kidney injury and moderate to severe ARDS. The manuscript has been amended to reflect this and this information has been added to Table 4.

How many patients received furosemide?

Following further review of the patient's medical records, only 11/55 (20%) received furosemide during the first 3 days of their ICU admission. This has been added to the manuscript.

Doxycycline was administered to patients with acute kidney injury?

We thank the reviewer for this question. Although we maintain a high index of suspicion for patients with leptospirosis in our area, it often takes several days or weeks to confirm this diagnosis microbiologically. Due to the presence of scrub typhus and Queensland tick typhus in our area that may present in a similar way to leptospirosis, we regularly use doxycycline empirically to cover several possible pathogens. We therefore commonly use doxycycline in people with acute kidney injury either as monotherapy or in combination with a beta-lactam antibiotic. Although doxycycline is commonly reserved for mild cases of leptospirosis, the excellent outcomes seen in our series suggest that there may be a role for this antibiotic in more severe cases, even in the context of acute kidney injury.

In addition, we have made a minor amendment to Table 7 of the manuscript.

Thank you once again, for considering our revised manuscript for publication in PLOS Neglected Tropical Diseases. If you require any further information or clarification, please do not hesitate to contact me.

Yours truly,

Dr Simon Smith

On behalf of the authors
